# Gene networks for use in metabolomic data analysis of blood plasma from patients with postoperative delirium

**DOI:** 10.18699/VJGB-23-89

**Published:** 2023-12

**Authors:** V.A. Ivanisenko, N.V. Basov, A.A. Makarova, A.S. Venzel, A.D. Rogachev, P.S. Demenkov, T.V. Ivanisenko, M.A. Kleshchev, E.V. Gaisler, G.B. Moroz, V.V. Plesko, Y.S. Sotnikova, Y.V. Patrushev, V.V. Lomivorotov, N.A. Kolchanov, A.G. Pokrovsky

**Affiliations:** Institute of Cytology and Genetics of the Siberian Branch of the Russian Academy of Sciences, Novosibirsk, Russia Novosibirsk State University, Novosibirsk, Russia Kurchatov Genomic Center of ICG SB RAS, Novosibirsk, Russia; Novosibirsk State University, Novosibirsk, Russia N.N. Vorozhtsov Novosibirsk Institute of Organic Chemistry of the Siberian Branch of the Russian Academy of Sciences, Novosibirsk, Russia; Institute of Cytology and Genetics of the Siberian Branch of the Russian Academy of Sciences, Novosibirsk, Russia; Institute of Cytology and Genetics of the Siberian Branch of the Russian Academy of Sciences, Novosibirsk, Russia Kurchatov Genomic Center of ICG SB RAS, Novosibirsk, Russia; Novosibirsk State University, Novosibirsk, Russia N.N. Vorozhtsov Novosibirsk Institute of Organic Chemistry of the Siberian Branch of the Russian Academy of Sciences, Novosibirsk, Russia; Institute of Cytology and Genetics of the Siberian Branch of the Russian Academy of Sciences, Novosibirsk, Russia Novosibirsk State University, Novosibirsk, Russia Kurchatov Genomic Center of ICG SB RAS, Novosibirsk, Russia; Institute of Cytology and Genetics of the Siberian Branch of the Russian Academy of Sciences, Novosibirsk, Russia Novosibirsk State University, Novosibirsk, Russia Kurchatov Genomic Center of ICG SB RAS, Novosibirsk, Russia; Institute of Cytology and Genetics of the Siberian Branch of the Russian Academy of Sciences, Novosibirsk, Russia; Novosibirsk State University, Novosibirsk, Russia N.N. Vorozhtsov Novosibirsk Institute of Organic Chemistry of the Siberian Branch of the Russian Academy of Sciences, Novosibirsk, Russia; E. Meshalkin National Medical Research Center of the Ministry of Health of Russian Federation, Novosibirsk, Russia; E. Meshalkin National Medical Research Center of the Ministry of Health of Russian Federation, Novosibirsk, Russia; Novosibirsk State University, Novosibirsk, Russia N.N. Vorozhtsov Novosibirsk Institute of Organic Chemistry of the Siberian Branch of the Russian Academy of Sciences, Novosibirsk, Russia Boreskov Institute of Catalysis of the Siberian Branch of the Russian Academy of Sciences, Novosibirsk, Russia; Novosibirsk State University, Novosibirsk, Russia Boreskov Institute of Catalysis of the Siberian Branch of the Russian Academy of Sciences, Novosibirsk, Russia; E. Meshalkin National Medical Research Center of the Ministry of Health of Russian Federation, Novosibirsk, Russia Penn State Health Milton S. Hershey Medical Center, Hershey, PA, USA; Institute of Cytology and Genetics of the Siberian Branch of the Russian Academy of Sciences, Novosibirsk, Russia Kurchatov Genomic Center of ICG SB RAS, Novosibirsk, Russia; Novosibirsk State University, Novosibirsk, Russia

**Keywords:** LC-MS/MS, metabolomics, lipidomics, postoperative delirium, cardiac surgery, biomarkers, sphingolipids, gene networks, ANDSystem, ВЭЖХ-МС/МС, метаболомика, липидомика, послеоперационный делирий, кардиохирургия, биомаркеры, сфинголипиды, генные сети, ANDSystem

## Abstract

Postoperative delirium (POD) is considered one of the most severe complications, resulting in impaired cognitive function, extended hospitalization, and higher treatment costs. The challenge of early POD diagnosis becomes particularly significant in cardiac surgery cases, as the incidence of this complication exceeds 50 % in certain patient categories. While it is known that neuroinflammation, neurotransmitter imbalances, disruptions in neuroendocrine regulation, and interneuronal connections contribute significantly to the development of POD, the molecular, genetic mechanisms of POD in cardiac surgery patients, along with potential metabolomic diagnostic markers, remain inadequately understood. In this study, blood plasma was collected from a group of patients over 65 years old after cardiac surgery involving artificial circulation. The collected samples were analyzed for sphingomyelin content and quantity using high-performance liquid chromatography coupled with mass spectrometry (HPLC-MS/MS) methods. The analysis revealed four significantly different sphingomyelin contents in patients with POD compared to those who did not develop POD (control group). Employing gene network reconstruction, we perceived a set of 82 regulatory enzymes affiliated with the genetic coordination of the sphingolipid metabolism pathway. Within this set, 47 are assumed to be regulators of gene expression, governing the transcription of enzymes pivotal to the metabolic cascade. Complementing this, an additional assembly of 35 regulators are considered to be regulators of activity, degradation, and translocation dynamics of enzymes integral to the aforementioned pathway. Analysis of the overrepresentation of diseases with which these regulatory proteins are associated showed that the regulators can be categorized into two groups, associated with cardiovascular pathologies (CVP) and neuropsychiatric diseases (NPD), respectively. The regulators associated with CVP are expectedly related to the effects on myocardial tissue during surgery. It is hypothesized that dysfunction of NPD-associated regulators may specifically account for the development of POD after cardiac surgery. Thus, the identified regulatory genes may provide a basis for planning further experiments, in order to study disorders at the level of expression of these genes, as well as impaired function of proteins encoded by them in patients with POD. The identified significant sphingolipids can be considered as potential markers of POD.

## Abstract (ru)

Послеоперационный делирий (ПОД) является серьезным осложнением, приводящим к нарушению когнитивных функций пациентов, увеличению длительности госпитализации, а также повышению расходов на лечение пациента. Проблема ранней диагностики ПОД приобретает особую важность в случае кардиохирургических операций, поскольку частота развития такого осложнения у некоторых категорий пациентов превышает 50 %. Известно, что в развитие ПОД большой вклад вносят нейровоспаление, дисбаланс нейромедиаторов, нарушение нейроэндокринной регуляции и межнейрональных связей, однако мо- лекулярно-генетические механизмы ПОД у пациентов, перенесших кардиохирургические операции, а также метаболомные диагностические маркеры, до сих пор плохо изучены. В данной работе с помощью метода высокоэффективной жидкостной хроматографии с масс-спектрометрической детекцией (ВЭЖХ-МС/МС) был проведен анализ содержания ряда сфингомиелинов в плазме крови пациентов старше 65 лет, взятой после операции на сердце в условиях искусственного кровообращения. Найдено четыре статистически значимо различающихся по содержанию сфингомиелина у пациентов с ПОД по сравнению с пациентами, у которых не развился ПОД (контрольная группа). С помощью реконструкции генных сетей, описывающих генетическую регуляцию пути метаболизма сфинголипидов, определены 82 регуляторных белка, из которых 47 – ре- гуляторы экспрессии генов, кодирующих ферменты метаболического пути, и 35 – регуляторы активности, деградации и транспорта ферментов данного пути. Анализ перепредставленности заболеваний, с которыми ассоциированы эти регуляторные белки, показал, что регуляторы можно разбить на две группы, ассоциированные с сердечно-сосудистыми патологиями и с нервно-психическими заболеваниями соответственно. Регуляторы, ассоциированные с сердечно-сосудистыми патологиями, ожидаемо связаны с воздействием на ткани миокарда во время операции. Сделано предположение, что нарушение функции регуляторов, ассоциированных с нервно-психическими заболеваниями, может специфически обусловливать развитие ПОД после кардиохирургической операции. Таким образом, выявленные регуляторные гены могут представлять основу для планирования дальнейших экспериментов по изучению нарушений на уровне экспрессии данных генов, а также нарушения функции кодируемых ими белков у пациентов с ПОД. Идентифицированные значимые сфинголипиды могут рассматриваться как потенциальные маркеры послеоперационного делирия

## Introduction

Postoperative delirium (POD) is a serious complication of the early postoperative period. Its incidence in cardiovascular surgery is 52 % (Brown, 2014). The development of POD leads to a worse prognosis, including longer hospitalization duration, increased complications and mortality, impaired cognitive function and physical status, and increased patient costs (Pisani et al., 2009; Gottesman et al., 2010). Delirium and postoperative cognitive impairment most commonly occur in patients over 60 years of age (Morimoto et al., 2009). Factors such as CNS hypoxia, embolism, neurotransmitter release, systemic inflammatory responses and other disorders, including metabolic issues, contribute to this phenomenon (Wimmer-Greinecker et al., 1998; Cerejeira et al., 2010).

Metabolomics is a branch of bioanalytical chemistry focused on the identification and quantification of low molecular weight metabolites (<1,500 Da). The metabolomic approach can be used to search for associations between metabolic signatures and disease phenotypes. In particular, metabolomic methods allow the detection of low molecular weight metabolites capable of crossing the blood-brain barrier, making metabolomic analysis a powerful tool for identifying markers of delirium (Ke et al., 2019). For example, several studies have shown that disturbances in energy metabolism, amino acid biosynthesis, omega-3 and omega-6 fatty acid deficiency, and glutamate-glutamine cycle dysfunction are associated with postoperative delirium in non-cardiac surgery (Guo et al., 2019; Tripp et al., 2021).

Previously, a number of our studies have shown the possibility of using the results of metabolomic screening in the search for biomarkers of pathologies, as well as the reconstruction of gene networks based on the obtained data. Thus, using statistical analysis of metabolomic profiles of cerebrospinal fluid (CSF) and blood plasma of patients with high-grade glioma obtained by HPLC-MS/MS, we have revealed correlations between metabolomic profiles of blood plasma and CSF (Rogachev et al., 2021). Metabolomic analysis combined with gene network reconstruction using ANDSystem to interpret metabolomic data (IvanisenkoV.A. et al., 2015, 2019; Ivanisenko T.V. et al., 2020, 2022) allowed us to identify key SARS-CoV-2 proteins whose interactions with human proteins could lead to impaired metabolic processes in COVID-19 patients (Ivanisenko V.A. et al., 2022).

Sphingomyelins (SM) are among the major phospholipids that make up the hydrophobic matrix of plasma membranes of mammalian cells; however, in response to stress, sphingomyelins can be cleaved by sphingomyelinase into phosphatidylcholine and ceramide, which have a signaling function. Changes in sphingomyelin metabolism can affect the balance of neurotransmitters in the brain, cause disruption neuronal connectivity, and induction of neuroinflammation, making them an important target for studying the mechanisms of delirium pathogenesis (Wang, Shen, 2018; Xiao et al., 2023).

In this study, the content of 9 phospholipids belonging to the sphingomyelin class in the plasma of patients undergoing cardiac surgery was analyzed using HPLC-MS/MS. There were 4 statistically significant different sphingomyelin contents in patients with POD compared with patients who did not develop POD (control group).

To explain possible mechanisms of sphingolipid metabolism disorders, we reconstructed gene networks describing the genetic regulation of the KEGG pathway “Sphingolipid metabolism” (hsa: 00600) using ANDSystem. Analysis of gene networks allowed us to identify 35 regulators of transport, activity and degradation of enzymes of this pathway, as well as 47 regulators of expression of genes encoding these enzymes.

## Materials and methods

Patients. The study included patients over 65 years of age who underwent cardiac surgery under artificial circulation. Exclusion criteria were: emergency intervention, aortic surgery, hemodynamically significant carotid artery stenosis, Parkinson’s disease, liver cirrhosis (Child-Pugh B or C), taking anticholinergic drugs, antidepressants, antiepileptic and chemotherapeutic drugs. Patients were recruited from June 2019 to January 2021. Atotal of 39 patients were included in the study (Table 1). Within 5 days after surgery, patients were evaluated for postoperative delirium using the CAM-ICU (Confusion Assessment Method for the Intensive Care Unit) test. The first test was performed 6–8 hours after surgery, and then the patients were assessed twice a day. Delirium was considered to be present if the CAM-ICU test was positive at least once.

The study was approved by the Ethics Committee of the E. Meshalkin National Medical Research Center (Novosibirsk, Russia).

Blood sampling and sample preparation. Blood samples were collected from patients 24 hours after cardiac surgery. Venous blood was collected into 10 mL BD Vacutainer® KEDTA tubes containing potassium EDTA as anticoagulant. Plasma was separated from blood cells by centrifugation for 15min at 2,000 g and +4 °C, separated into aliquots and stored frozen at –80 °C until further use.

All samples were processed simultaneously according to the protocol described in (Li et al., 2017): 400 μL of a chilled mixture of methanol and acetonitrile (1:1) was added to 100 μL of blood plasma. The samples were shaken on a shaker, then centrifuged for 15 min at +4 °C and 16,000 rpm. The supernatant was transferred to a glass vial insert and analyzed. Two quality control samples, obtained by mixing equal volumes of plasma samples from patients with POD and controls, were prepared using the same procedure.

HPLC-MS/MS analysis was performed on a Shimadzu LC-20AD Prominence chromatograph equipped with a gradient pump, SIL-20AC autosampler (Shimadzu, Japan), thermostated at 10 °C, and CTO-10ASvp column thermostat, with a temperature of 35 °C. Chromatographic separation was carried out on a monolithic column with 1-vinyl-1,2,4-triazole based sorbent (Basov et al., 2024). The monolithic material was synthesized in glass tubes with an inner diameter of 2 mm as described previously (Patrushev et al., 2020). Mobile phaseA was an aqueous 20 mM ammonium carbonate solution adjusted to pH = 9.8 with 25 % aqueous ammonia solution and containing 5 vol % acetonitrile; mobile phase B was pure acetonitrile. The elution gradient was as follows: 0 min – 0 % B, 1 min – 0 % B, 6 min – 98 % B, 16 min – 98 % B, after which the column was equilibrated for 3 min. The flow rate was 300 μL/min and the sample volume was 2 μL.

Metabolites were detected on an API 6500 QTRAP mass spectrometer (AB SCIEX, USA) equipped with an electrospray ionization source operating in positive ionization mode. Metabolites were detected in multiple reaction monitoring (MRM) mode.

The main mass spectrometric parameters were as follows. The voltage at the ion source was 5500 V. The dryer gas temperature was 475 °C, CAD gas pressure was “high”, GS1, GS2 and curtain gas pressures were 33, 33 and 30 psi, respectively. The declustering potential (DP) was 91 V, the entry potential (EP) was 10 V, and the collision cell exit potential (CXP) was 10 V. The precursor and fragment ion transitions, metabolite names, residence times, and corresponding collision energies are presented in the Supplementary Table S11. Instrument control and data acquisition were performed using Analyst 1.6.3 software (AB SCIEX, Framingham, MA, USA). Chromatograms were processed using the MultiQuant 2.1 program (AB SCIEX, USA).


Supplementary Materials are available in the online version of the paper: https://vavilovj-icg.ru/download/pict-2023-27/appx24.xlsx


**Table 1.**
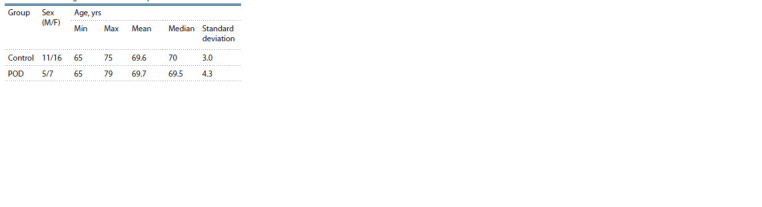
Sex and age characteristics of patients

Data preprocessing and statistics analysis. The raw data were preprocessed to fill in missing values of metabolite content in the analyzed samples as follows. If the number of samples with missing values did not exceed 5 % of the total number of values for 39 patients, the median was calculated for the remaining samples and taken as the metabolite content value. This approach is due to the robustness of the median to outliers. Statistical differences in the content of metabolites in blood plasma samples in the group of patients with and without POD were assessed using the nonparametric Mann– Whitney criterion.

Reconstruction and analysis of gene networks. The list of genes encoding enzymes involved in the “Sphingolipid metabolism” pathway (ID: hsa00600) was extracted from the KEGG database (https://www.kegg.jp/kegg/pathway.html, Kanehisa, 2002; Kanehisa et al., 2022). The regulatory gene network was reconstructed using the ANDSystem software and information system (Ivanisenko V.A. et al., 2015, 2019; IvanisenkoT.V. et al., 2020, 2022). Work with the ANDSystem knowledge base was performed using the ANDVisio program module. Analysis of overrepresentation of biological processes (Gene Ontology) and diseases associated with regulatory gene network proteins was performed using the web-based tool DAVID (https://david.ncifcrf.gov/tools.jsp, Huang D.W. et al., 2009).

## Results

Study of sphingolipid content in patients’ blood plasma using the HPLC-MS/MS method

Since impaired sphingomyelin metabolism may contribute to the development of delirium, the aim of our analysis was to investigate their role in the complication of POD by examining their content in the plasma of patients after cardiac surgery. Specifically, we comparatively analyzed SM expression in the plasma of patients undergoing cardiac surgery. The metabolites of this class that had a significant statistical difference in content within the samples taken from the group of patients with POD were compared with those from the group of patients who did not develop POD and are summarized in Table 2.

**Table 2.**
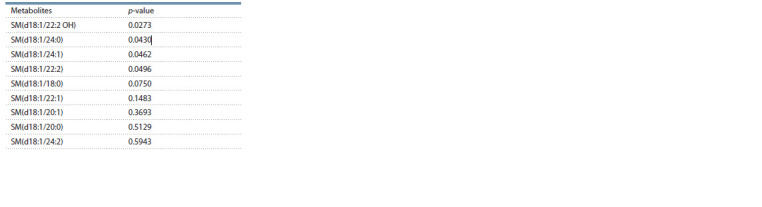
Statistical significance of differences between the group of patients with POD and the control group in the content of metabolites in blood plasma samples when compared by the Mann–Whitney test

According to the Mann–Whitney test, out of these 9 sphingomyelins analyzed, four (SM(d18:1/22:2 OH), SM(d18:1/24:0), SM(d18:1/24:1), and SM(d18:1/22:2)) showed statistically significant (p-value <0.05) differences between the studied patient groups. We hypothesized that the impaired metabolism of sphingolipids may be related to the impaired metabolic pathway of their biosynthesis. To test this hypothesis, using the ANDSystem software and information system, we reconstructed and analyzed the gene network describing the regulation of expression of genes encoding enzymes of the KEGG “Sphingolipid metabolism” metabolic pathway, as well as the regulation of transport, activity, and degradation of these enzymes.

Reconstruction of the regulatory gene network

To reconstruct the regulatory gene network, a list of genes encoding enzymes involved in sphingolipid metabolism “Sphingolipid metabolism” (hsa00600) was extracted from the KEGG database. The resulting list contained 43 human genes (Supplementary Table S2). The gene network graph was reconstructed in the “Query Wizard” module of ANDVisio.

It should be noted that in the gene network we considered only regulatory connections directed from regulatory proteins to enzymes of the metabolic pathway. The resulting gene network contained 43 human genes, 125 proteins (43 metabolic pathway enzymes and 82 regulatory proteins), and 159 interactions between them (see the Figure). Different types of interactions between gene network members were represented in the following ratio: 28 links corresponding to the type “regulation of activity”, 2 – “regulation of degradation”, 4 – “proteolysis”, 8 – “regulation of transport”, 43 – “expression”, 74 – “regulation of expression”.

**Fig. 1.**
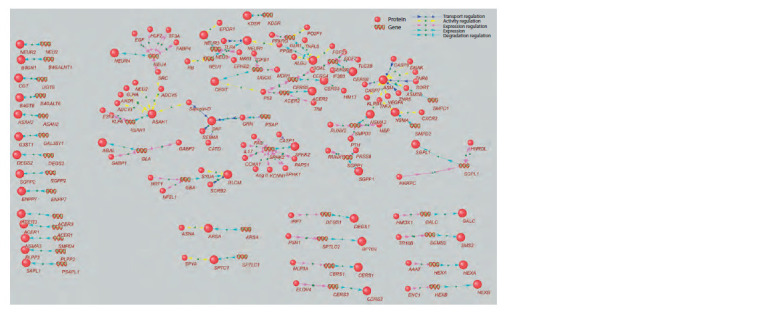
Gene network regulation of the sphingolipid metabolism pathway.

To investigate the association of regulatory proteins with pathologies, we analyzed the overrepresentation of diseases and biological processes by Gene Ontology, using the webbased tool DAVID. A list consisting of 82 genes encoding gene-network regulatory proteins was provided as the input. The results of the disease and biological processes overrepresentation analysis are summarized in Supplementary Tables S3 and S4, respectively.

All regulatory proteins represented in the gene network (see the Figure) can be divided into two groups: (1) regulators of gene expression and (2) regulators of activity, stability, transport, etc., which can be called regulators of protein function. To investigate the features associated with these diseases and biological processes, overrepresentation analysis was performed separately for each of these groups of proteins (Supplementary Tables S5–S8).

## Discussion

According to the literature, sphingomyelins (SM) play an important role in nervous system function, and alterations in their metabolism may contribute to the development of delirium by inducing neuroinflammation, altering neurotransmitter balance, and disrupting neuronal connectivity (Wang, Shen, 2018; Xiao et al., 2023). Our metabolomic analysis using HPLC-MS/MS of blood plasma from patients undergoing cardiac surgery allowed us to identify 4 out of 9 sphingomyelins, the content of which had a significant statistical difference in the analyzed samples of patients with POD compared to patients who did not develop POD (see Table 2).

To study potential mechanisms of sphingolipid metabolism disorders, a gene network (see the Figure) describing the regulation of gene expression and function of the enzymes encoded by them, participants of the KEGG metabolic pathway “Sphingolipid metabolism” (Sphingolipid metabolism, hsa: 00600), was reconstructed using ANDSystem. Network analysis showed that 82 regulatory proteins were involved in the regulation of the metabolic pathway, the dysfunction of which could influence the impairment of sphingolipid metabolism. Based on the enrichment analysis of the list of genes encoding these proteins with disease-associated genes, 168 statistically significantly overrepresented diseases were identified.

To simplify the presentation of the results, the list of diseases was divided into five groups (Table 3). The most significant disease was from the group of cardiovascular system pathologies, which may be due to the fact that all patients underwent cardiac surgery due to cardiac pathologies. The surgeries and medical procedures performed, such artificial circulation, may also explain the presence of the groups “inflammation”, “renal pathologies” and “surgical intervention” among the identified significant pathologies (Stafford-Smith et al., 2008; Squiccimarro et al., 2019). These pathologies could be concurrently associated with both groups of patients, with and without POD, as each had undergone cardiac surgery.

**Table 3.**
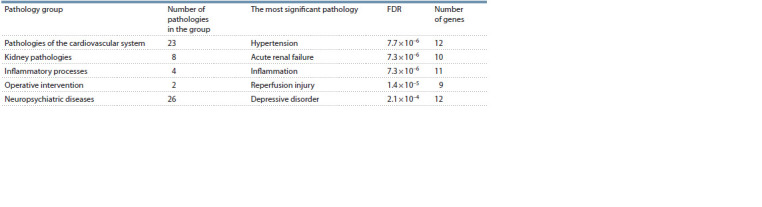
Statistical significance of disease overrepresentation based on gene-regulatory list analysis False Discovery Rate (FDR) and the number of genes associated with the pathology are given for the most statistically significant pathology.

The “neuropsychiatric diseases” group of pathologies is of particular interest in the context of the development of postoperative delirium. In particular, H. Huang et al. (2022) discuss the role of neuroinflammation in the development of postoperative delirium. The authors emphasize neuroinflammation and disruption of the blood-brain barrier as some of the main pathophysiological factors in the onset of delirium. The association of neuropsychiatric pathologies with POD is also widely discussed in the scientific literature. For example, O’Sullivan et al. consider that the link between delirium and depressive disorder may be due to common pathophysiological mechanisms including impaired stress and inflammatory responses, monoamine and melatoninergic signaling (O’Sullivan et al., 2014). According to our analysis, at the molecular genetic level, these pathophysiologic mechanisms may involve genetic regulation of the sphingolipid metabolism pathway. The list of regulatory genes from the gene network associated with the “neuropsychiatric diseases” group is summarized in Supplementary Table S9

Statistical analysis of Gene Ontology overrepresentation of biological processes based on the list of regulatory genes identified 67 significant biological processes (BPs, Supplementary Table S4). The list of biological processes was categorized into 7 groups to represent the results (Table 4). Among the significant BPs were found fundamental regulatory processes, including regulation of transcription, regulation of proliferation, activation of cell signaling pathways, etc. These results were expected because the gene network members are regulators of gene expression and functions of the enzymes they encode. The set of regulators we analyzed turned out to be enriched with genes involved in the process of cardiovascular cell proliferation (see Table 4 and Supplementary Table S10), which can be explained by the activation of regenerative processes after surgical intervention. In addition to those mentioned above, it is possible to identify more specifically delirium-related BPs, such as inflammatory processes, response to stress factors and regulation of apoptosis (Steiner, 2011; Vutskits, Xie, 2016).

**Table 4.**
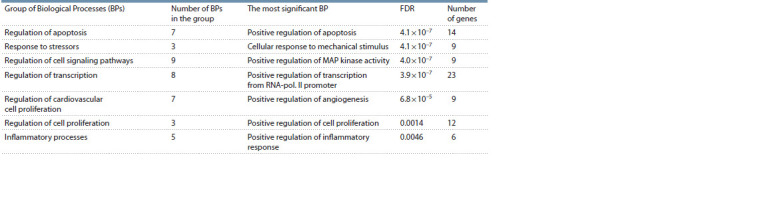
Statistical significance of overrepresentation of biological processes based on gene-regulatory list analysis False Discovery Rate (FDR) and the number of genes associated with BPs are given for the most statistically significant BPs.

Notably, the regulatory connections in the gene network can be divided into two groups: regulators of the gene expression and regulators of the functions (activity, degradation, and transport) of protein products of their expression. It is of interest to consider whether there are characteristic features related to the molecular mechanisms of delirium development for regulators from these two separate groups. We analyzed the overrepresentation of diseases and biological processes separately for regulators of expression as well as for regulators of protein function (see Supplementary Tables S5–S8)

Unexpectedly, when considering regulators of protein function, the top ten most significant pathologies were neuropsychiatric diseases (e. g., schizophrenia, bipolar disorders, autism), which, according to the literature, are specifically associated with delirium (García-Bueno et al., 2016a, b). Interestingly, the literature discusses the association of preoperative pain factors with depressive symptoms and the subsequent development of POD (O’Sullivan et al., 2014). When considering the regulators of gene expression, among the significant pathologies, the group of pathologies of the cardiovascular system was predominant, which was expected, given the patients’ history. In this regard, it can be assumed that a special role in the manifestation of pathological mechanisms of delirium belongs to the regulation of the activity of protein products and, to a lesser extent, to the regulation of gene expression. Note that no significant differences between the two groups of regulators were found as a result of the analysis of BP overrepresentation.

An important structural characteristic of the graph of gene networks, which determines the peculiarities of their functioning, is the centrality of vertices. One of its indicators is the degree centrality of vertices, which characterizes the ratio of the number of links of a given vertex to the total number of links in the graph and is widely used in the analysis of gene networks. The enzyme sphingomyelinase (ASM, see the Figure) had the largest number of connections (regulation of activity, degradation, and transport) with regulatory proteins among the graph vertices corresponding to enzymes. This enzyme cleaves sphingomyelins into phosphatidylcholine and ceramide, which have a signaling function. The function of ASM enzyme was modulated by 10 regulatory proteins, 6 of which had the “activity regulation” type of links (ASM3B, Hsp70, KLRB1, TNFA, TNR6, VEGFA), 3 proteins (CASP8, SORT, TNR5) had the “transport regulation” type, and there was one link with CASP7 protein with the “proteolysis” type. Note that among the regulatory proteins, caspase-8 (CASP8) and tumor necrosis factor alpha (TNFA) were present and found to be associated with overrepresented diseases such as epilepsy, depression, dementia and other neuropsychiatric diseases. According to the literature, CASP8 accomplishes the activation and translocation of ASM to the surface of the plasma membrane. ASM activation results in the cleavage of sphingomyelins and the formation of ceramide, which promotes caspase-8 activity and induction of apoptosis (Grassmé et al., 2003). Surgical interventions are known to provoke the penetration of pro-inflammatory factors such as interleukins and TNFA across the GEB, which contributes to neuroinflammation and may be associated with the development of POD (Alam et al., 2018). According to the reconstructed gene network, TNFA increases phosphomyelinase activity (Corre et al., 2013) and is also associated with overrepresented neuropsychiatric diseases such as depression, epilepsy, etc. (see Supplementary Table S3).

The SPHK2 gene (see the Figure), encoding the enzyme sphingosine kinase 2, had the highest centrality index among the gene network graph nodes corresponding to the genes. The gene network represented 7 regulators of expression of this gene encoded by the AGT, CCNA1, FAS, IL17A, KCNN1, SPHK1, and PAPSS1 genes (see the Figure). In contrast to the peak corresponding to the ASM protein, there were no regulators of SPHK2 expression associated with neuropsychiatric diseases. This fact once again indicates that the most important contribution to the dysfunction of the sphingolipid metabolism pathway associated with postoperative delirium may come not from the regulation of the expression of genes encoding enzymes of the metabolic pathway, but from the impaired transport, activity, and stability of the products of these genes. Genes associated with other disease groups were represented among the regulators of SPHK2 expression (see Supplementary Table S5). For example, fatty acid synthase (FAS) activity is associated with myocardial infarction, hypertension, type II diabetes, and other diseases (Nosrati-Oskouie et al., 2021).

## Conclusion

An integrated approach in metabolomic analysis of blood plasma from cardiac surgery patients using HPLC-MS/MS and bioinformatic methods of ANDSystem gene network reconstruction allowed us to identify potential markers of the sphingomyelin class, as well as regulatory genes, the dysfunction of which may underlie the mechanisms of postoperative delirium (POD) development. The analysis of disease overrepresentation revealed that the groups of pathologies such as neuropsychiatric diseases, cardiac and renal pathologies, inflammatory processes, and surgical intervention were associated with these regulatory proteins. The function of regulators associated with CVDs could be impaired in patients with POD due to heart surgery and medical procedures such as artificial circulation (Gao et al., 2005). However, since heart surgery was undergone by all subjects, it can be expected that the altered function of these regulatory proteins could have equally affected both the group with and without POD. In this regard, the function of a group of regulators associated with neuropsychiatric diseases could have been specifically impaired in patients with POD, which was responsible for the decreased plasma sphingolipid content in these patients.

Among the nodes of the gene network graph, the node with 10 regulatory connections corresponding to the ASM enzyme (phosphomyelinase) had the highest centrality index. Proteins encoded by the TNFA, CASP8, TNR5, and VEGFA genes, which are associated with epilepsy, depression, and other neuropsychiatric diseases, were found among regulators of ASM activity and transport. Among the nodes corresponding to the genes, the SPHK2 (sphingosine kinase 2) gene had the highest centrality score in the graph. The expression of this gene is regulated by 7 proteins encoded by the AGT, CCNA1, FAS, IL17A, KCNN1, SPHK1, and PAPSS1 genes.

The proposed hypotheses on the role of regulatory genes in the development of AMP can be used to plan transcriptomic and proteomic analysis experiments to study the molecular genetic mechanisms of this complication.

## Conflict of interest

The authors declare no conflict of interest.
